# Impact of Striped-Squirrel Nectar-Robbing Behaviour on Gender Fitness in *Alpinia roxburghii* Sweet (Zingiberaceae)

**DOI:** 10.1371/journal.pone.0144585

**Published:** 2015-12-21

**Authors:** Xiaobao Deng, Dharmalingam Mohandass, Masatoshi Katabuchi, Alice C. Hughes, David W. Roubik

**Affiliations:** 1 Key Laboratory of Tropical Forest Ecology, Xishuangbanna Tropical Botanical Garden (XTBG), Chinese Academy of Sciences, Menglun, Mengla County, Yunnan– 666 303, P.R. China; 2 Department of Biology, University of Florida, 322 Carr Hall, Gainesville, FL, 32611, United States of America; 3 Centre for Integrative Conservation, Xishuangbanna Tropical Botanical Garden (XTBG), Chinese Academy of Sciences, Menglun, Mengla County, Yunnan– 666 303, P.R. China; 4 Smithsonian Tropical Research Institute, APDO 0843–03092, Balboa, Ancón, Republic of Panama; University of Northampton, UNITED KINGDOM

## Abstract

Nectar-robbing has the potential to strongly affect male and female reproductive fitness of plants. One example of nectar theft is that shown by striped-squirrels (*Tamiops swinhoei*) on a number of ginger species, including *Alpinia roxburghii* and *A*. *kwangsiensis* (Zingiberaceae). In this study, we used a fluorescent dye as a pollen analogue, and measured fruit and seed output, to test the effect of squirrel nectar-robbing on *A*. *roxburghii* reproductive fitness. Pollen transfer between robbed and unrobbed flowers was assessed by comparing 60 randomly established plots containing robbed and unrobbed flowers. The frequency of squirrel robbing visits and broken styles were recorded from a number of flowers for five consecutive days. Two bee species (*Bombus eximius* and *Apis cerana)*, were the primary pollinators, and their visitation frequency was recorded for six consecutive days. The results showed that fluorescent powder from unrobbed flowers was dispersed further, and to a greater number of flowers than that placed on robbed flowers. Additionally, robbing flowers caused significant damage to reproductive organs, resulting in lower fruit and seed sets in robbed than in unrobbed flowers and influencing both male and female fitness. The frequency of the primary pollinator visits (*B*. *eximius*) was significantly higher for unrobbed plants than for robbed plants. The present study clearly shows the negative impact of squirrel robbing on *A*. *roxburghii* male reproductive fitness and neutral impact on female reproductive fitness.

## Introduction

Many previous studies have shown that legitimate pollinators and nectar robbers display different behaviours and which may influence plant reproductive success. Nectar robbers including insects, birds and mammals, generally make a hole at the base of the flower corolla through which they extract nectar without pollinating the flowers [1–4]. Several studies have shown that insect nectar robbing behaviour indirectly affects plant floral parts, while nectar robbing directly affects plant reproductive success [5–7]. For instance, bird nectar robbing behaviours substantially damages plant floral parts [8] as birds use their beak to make a hole through the standing crop of flowers [8–10]. Similarly, small mammals like the striped-squirrel *(Tamiops swinhoei)* usually damage the flower style while robbing nectar [11]. These robbing behaviours significantly reduced fruit and seed set and thus have considerable negative impacts on male and female plants reproductive fitness [8, 11].

Several studies have demonstrated that nectar robbers also differentially influence male and female plant reproductive fitness [7, 12–13]. Moreover, once floral parts are damaged, pollinators may react to this damage by reducing their visitation rates and changing their flight distance [7–8, 14]. Additionally, nectar robber activity influences the number of seeds sired, pollen removal and pollen donation (assessed using fluorescent dye as a pollen analogue)[6–7, 15–16]. Studies quantifying pollinator flight distances after robbed versus unrobbed flower visitation [7, 13, 17] found that robbed flowers displayed decreased nectar availability causing subsquent alterations in the required inter-plant flight distances by pollinators [18–19] As a result of the reduced nectar rewards, there is also likely to be increased pollen flow distances [17, 19], a reduction in the number of flowers or inflorescences visited per patch [5, 20–22] and a reduced time spent per flower visitation [17, 23] Previous nectar robbing studies demonstrated that nectar robbing by insects, birds and mammals substantially affected female reproductive success including fruit and/or seed set [2, 7, 12, 15–16, 18, 24–26]. Examining the variability in pollinator visitation, pollen deposition and pollen limitation is important to predict the effects of robbing on female reproductive fitness [27–29]. Self-compatible plants that cannot autogamously self-pollinate highly rely on pollinators for reproduction, such nectar robbing behaviours may affect plant reproduction. Moreover, since nectar robbing decreases the amount of available nectar on flowers and the frequency of pollinator visitation, plant female reproduction is substantially affected [28–30]. However, the impacts of nectar-robbing on plant reproductive fitness can vary considerably. For example, some studies have reported positive, negative and neutral effects, depending on the plant and nectar robber species examined [3, 16–18, 20].

The majority of the previous studies identified insects and birds as the most common nectar robbers in tropical and temperate habitats [2, 11, 13, 18]. However, the impacts of mammal nectar robbers on female plant fitness have rarely been studied [11] and no studies have previously examined impacts on male plant fitness. Here, we focused on the impact of nectar-robbing by the striped-squirrel, *Tamiops swinhoei* on male and female plant reproductive fitness. The striped-squirrel is a a major mammal nectar-robber of the self-compatible shrub species, *Alpinia roxburghii (*Zingiberaceae). Nectar-robbing by squirrels has previously been studied in a congeneric ginger species, *A*. *kwangsiensis* [11]. In that study, Deng *et al* [11] found a significant decrease in the fruit-set of flowers robbed by the squirrel *Tamiops swinhoei*, through negative physical impacts (through direct floral damage) which resulted in decreased female reproductive success [11]. However, total seed set was not included in the above study, which is unfortunate as assessing the changes in seed set can aid in understanding the impacts of robbery on female reproductive fitness. In addition, according to our field observations and video recordings, striped-squirrels do not consume flowers, but only consume nectar, often damaging the flowers and style in the process. To extract nectar, squirrels break the pedicel, remove the flower, perforate the corolla base and then drink the nectar. Conversely, although squirrels act as pollinators for some plants [31, 32], the net impact of squirrel nectar robbing on plant fitness has rarely been studied. We therefore address the following questions: (i) How does pollen transfer distance differ between robbed and unrobbed plots? (ii) How does fruit and seed production change between robbed and unrobbed plots? (iii) Does striped-squirrel robbing behaviour significantly affect floral reproductive parts? (iv) How does squirrel robbing behaviour impact plant fitness?

## Materials and Methods

### Study area and organisms

The study was carried out in a monsoon evergreen broad-leaf forest in the Caiyanghe Nature Reserve (22°30' N, 101°2' E; 1200 m asl), Yunnan Province, southwest China. The study site is part of the Xishuangbanna Station for Tropical Rain Forest Ecosystem Studies and Caiyanghe National Park run by the Chinese Forestry Administration. The field experiments were conducted during 2005–2007, from March to April. The populations of *A*. *roxburghii* occur naturally within several valleys, which contain evergreen broad-leaf forests whose canopy is primarily dominated by *Betula alnoides* Buch.-Ham. ex D.Don and *Alnus nepalensis* D.Don., [33]. In the study area, *A*. *roxburghii* is the dominant understory shrub.

**Fig 1:**
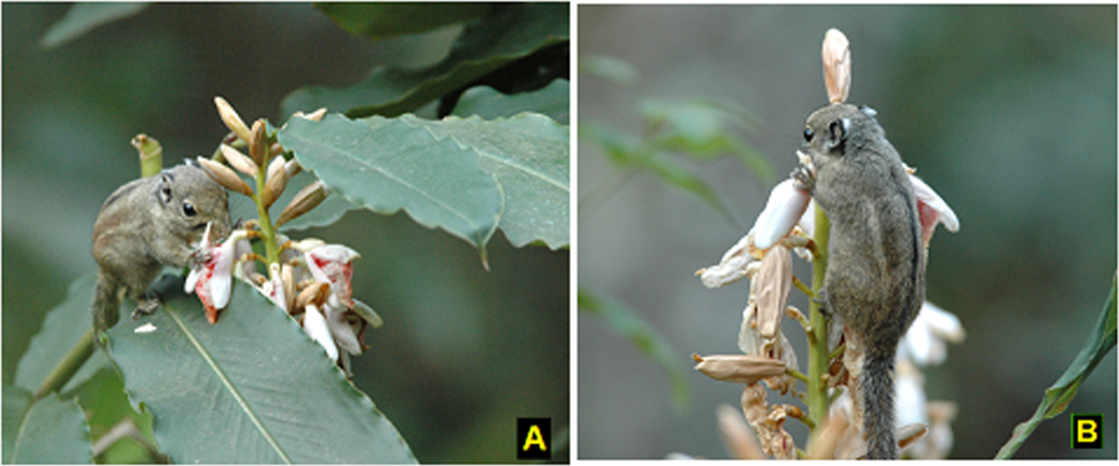
(A) A major nectar-robber, striped-squirrel (*Tamiops swinhoei*) robbing the nectar of *A*. *roxburghii* flowers, showing damage to the flower and the broken style. (B) Striped-squirrel makes a hole at the base of the flower corolla and sucks the nectar, through this behaviour squirrel damages the flowers.

According to the APG III plant classification, *Alpinia roxburghii* (Sweet) is a synonym of *Alpinia blepharocalyx* K. Schum. (family; Zingiberaceae) [34]. It is a self-compatible, hermaphroditic, clonal, flexistylous perennial herb, usually 1–3 m tall [33, 35]. The inflorescences are terminal on leafy shoots and 20–30 cm long. Bracteoles are green and elliptic, dry and brittle, and fall off at anthesis. During flowering, each inflorescence produces 2–10 open flowers per day, each lasting one day only [35]. Flowers produce nectar throughout the day, from 6 AM to 8 PM [33]. Flowering generally occurs from March to late April and capsules are mature by August–September [33, 35]. The flower has a peculiar structure: a conspicuous three-lobed labellum produced by the fusion of two staminodes, which are flesh-colored and red with a yellow center (Fig 1A). The labellum forms a tube, the free part of which is extended and forms a landing platform for pollinators. Only one fertile stamen with two anthers develops, and the style extends through the anthers. The base of the style is fixed within a groove in the filament, yet its apical quarter first flexes upward, then downward (or vice versa), hence the term ‘flexistyly’. The stylar movements in the two floral morphs are synchronous and correspond to foraging behaviour of insect pollinators such as *Bombus eximius* Smith and *Apis cerana* Fabricius (Apidae). This stylar movement characteristic indicates that reducing self-pollination may be a primary function of different floral morphs [33, 36].

*Tamiops swinhoei hainanus* Allen (Sciuridae; Swinhoe’s striped squirrel), the striped-squirrel, is a small-bodied squirrel *ca*. 10 cm in length and 60 g in weight. Its general dorsal color is olive-gray, with grey flanks, while the inner light stripes are pale yellowish-brown and extending to the tail base (Fig 1A and 1B) [11]. The species is easily recognized because of its characteristic light yellow stripes extending from nose to neck, and the white tufted hair on the posterior tip of the ears. The striped-squirrel is widely distributed in southern Asia and is very common in the Caiyanghe Nature Reserve, southern China [11]. Squirrels are primarily seed dispersers or seed predators and known to feed on fruits, seeds and insects [37, 38]. No sampling or specimen collection of squirrels occurred as part of this study.

### The effects of nectar robbing on pollen flow

**Fig 2:**
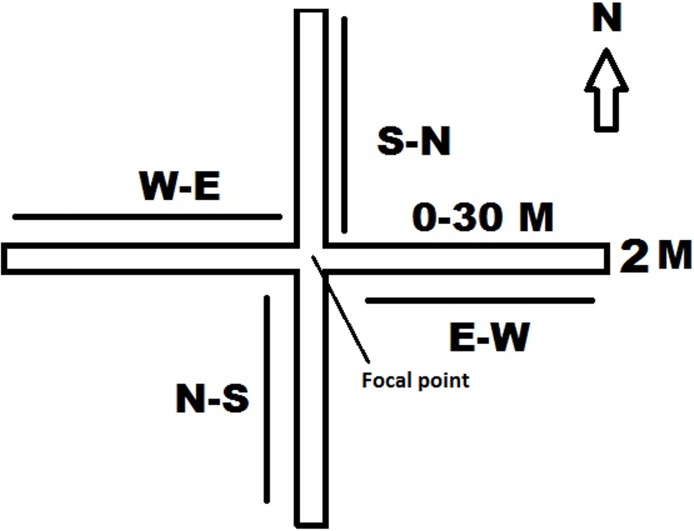
A model of the four-direction plots (FDP) established in a large population of *A*. *roxburghii* to compare robbed and unrobbed flowers.

To assess the effects of nectar robbing on pollinator flight between flowers, fluorescent powder (Radiant Color, Richmond, CA, USA) [7] was used as a pollen analogue [39], as dye transfer resembles pollen transfer by pollinators [7, 40]. We used a two by Four-Directional Plot (FDP) sampling design (for robbed and unrobbed treatments) (Fig 2). Four-directional plots were established laid by extending four transect lines of 2 m^2^ wide × 30 m long laid West to East (W-E), North to South (N-S), East to West (E-W), and South to North (S-N) (Fig 2). In the FDP, every 2 m^2^ interval was considered as a subplot. Additionally, every 30 m transect had 15 subplots and thus each FDP had 60 subplots, giving 120 subplots when both robbed and unrobbed plots are considered. We used the FDP design for this study in order to measure pollen flow distance across different cardinal directions. The distance between robbed and unrobbed plots was approximately 300 m and within the same population of plants. However, these two plots were not parallel to each other. After we laid the two plots separately, we marked a 2 m^2^ focal point in each plot within the FDPs (Fig 2). At the focal point of both the robbed and unrobbed plots, we selected three independent flowering branches of same *A*. *roxburghii* individual and applied fluorescent dye. Three independent flowering branches were selected, as *A*. *roxburghii* is a tufted plant usually producing many branches but only a single inflorescence per branch. We then covered the entire inflorescence in a mesh bag measuring 20 × 30 m, one day prior to flower opening. The next day, we marked 3–9 newly opened flowers and applied approximately 5 mg of fluorescent powder to the inside of each flower. We used a different fluorescent color (such as red, green, blue and yellow) each day to ensure the accurate calculation of the frequency of pollinator visits. Each day, we applied the fluorescent powder before 8 AM. Consequently, three focal inflorescences in both robbed and unrobbed FDP were marked for eight consecutive days, allowing us to observe pollen flow distance in each of the four directions. On the same day of the fluorescent powder application, we checked for the presence of the powder at 2 m^2^ intervals by using a UV-fluorescent flashlight in the evening after 7 PM. The unrobbed FDP was artificially maintained, by observers who actively prevented potential robbers by staying onsite until 8 PM each day. We also carefully observed the covered inflorescences and ensured that fluorescent-dyed flowers did not deter nectar robbers, as squirrels could easily remove or destroy them during the observation period. Thus, by having four observers guarding the unrobbed FDP, we effectively controlled nectar robbing in those patches. Based on direct field observations, we noted that, the squirrels do not visit the studied species during the night to rob nectar.

### Fruit and seed set

In 2005 and 2007, during the peak fruiting season, we randomly selected 60 inflorescences for fruit collection in both robbed and unrobbed FDPs. We counted seeds from each of the collected fruits. In 2005, we collected 2226 fruits from 9111 marked flowers and 3423 fruits from 9317 marked flowers in each 60 inflorescences from in robbed and unrobbed FDPs respectively. Similarly, in 2007, we collected 2948 fruits from 8120 marked flowers and 3578 fruits from 7528 marked flowers from each of the 60 inflorescences in robbed and unrobbed FDPs respectively. In each inflorescence, flower number ranged from 6–86 in robbed and 11–77 in unrobbed FDPs over the two study years 2005 and 2007. Seeds were counted from 684 collected fruits in 2005 and in 2007 and seeds were counted from a total of 1040 fruits in the robbed and unrobbed FDPs separately (S2 Table).

### Striped-squirrel visitation

Striped-squirrel identification and robbing behaviours were quantified through direct observations and video recordings. We recorded the number of robbed flowers and the resulting broken styles or flowers picked during 20 h over five consecutive days. In 2006, a total of 666 flowers from 50–60 inflorescences were observed for five consecutive days to record squirrel visits. Per observation day, 78–176 flowers were observed for 4 hours to record the number of visits/day.

### Pollinator visitation

In an earlier study, eight species of insects and birds were recorded as visitors of *A*. *roxburghii* [41], and among those, *Bombus eximius* and *Apis cerana* were the most common. Therefore, we observed flower visitation frequency of *Bombus eximius* and *Apis cerana* for a 4 hour time period per day over six consecutive days in both the robbed and unrobbed FDPs.

### Statistical analyses

We developed hierarchical Bayesian models to analyse effects of robbing on floral parts. One of the advantages of using hierarchical Bayesian modeling for the analysis is that we can estimate a large number of types of effects simultaneously (i.e., fixed effects, and the random effects of time and space, which are auto-correlated).

#### Pollen dispersal

We modeled the number of flowers with fluorescent dye (FD), in relation to the number of opened flowers (O) as a function of robbing effects (ROBBED: two levels, robbed and unrobbed) and distance effects (DISTANCE: eight levels, 0–2 m, 4–6 m, 8–10 m, 12–14 m, 16–18 m, 20–22 m, 24–26 m, 28–30 m) as follows:

$${FD}_{i}=Binomial\left(p_{i}^{\left(FD\right)},O_{i}\right),$$

$$logit\left(p_{i}^{\left(FD\right)}\right)=\beta_{1}^{\left(FD\right)}+{\beta_{2}^{\left(FD\right)}\times ROBBED}_{i}+\Sigma \left(\beta_{m}^{\left(FD\right)}\times {DISTANCE}_{i}\right)+r_{j}^{\left(FD\right)}+s_{k}^{\left(FD\right)}$$

The expressions above include a random effect for sampling date *j*, where *r* ^*(FD)*^_*j*_ ~ Normal (0, σ_FD r_), and for plot *k*, where *s* ^*(FD)*^_*k*_ ~ Normal(Σ*μ* ^*(FD)*^/*n*_*k*_, σ_FD s_), *μ* ^*(FD)*^ is the expected values for the plots adjacent to plot *k*, and *n*_*k*_ is the number of plots adjacent to plot *k*. The terms σ_FD r_ and σ_FD s_ represent the standard deviation of *r* ^*(FD)*^_*j*_ and *s* ^*(FD)*^_*k*_, respectively. The random plot effect was incorporated by using the conditional autoregressive model [42] to control for spatial autocorrelation in the probability of pollen deposit within quadrats.

#### Fruit and seed set

We modeled the number of flower fruit sets (F) in relation to the number of flowers (N) in inflorescence *i* as a function of robbing effects (ROBBED: two levels, robbed and unrobbed), as follows:

$$F_{i}=Binomial\left(p_{i}^{\left(F\right)},N_{i}\right),$$

$$logit\left(p_{i}^{\left(F\right)}\right)=\beta_{1}^{\left(F\right)}+\beta_{2}^{\left(F\right)}\times {ROBBED}_{i}+r_{j,k}^{\left(F\right)}$$

The expressions above include random effects for sampling plot *j* and year *k* simultaneously, where *r* ^*(F)*^_*j*,*k*_ ~ Normal (0, σ_F r_). The term σ_F r_ represents the standard deviation of *r* ^*(F)*^_*j*,*k*_.

We modeled the number of seed sets (S) of inflorescence *i* as a function of robbing effects (ROBBED: two levels, robbed and unrobbed), as follows:

$$S_{i}=Poisson\left(\lambda_{i}^{\left(S\right)}\right),$$

$$log{(\lambda }_{i}^{\left(S\right)})=\beta_{1}^{\left(S\right)}+\beta_{2}^{\left(S\right)}\times {ROBBED}_{i}+r_{j,k}^{\left(S\right)}$$

The expressions above include random effects for sampling plot *j* and year *k* simultaneously, where *r* ^*(S)*^_*j*,*k*_ ~ Normal (0, σ_S r_). The term σ_S r_ represents the standard deviation of *r* ^*(S)*^_*j*,*k*_.

Robbing effects on styles

We modeled the number of robbed flowers (ROBBED) in inflorescence *i* as a function of the number of flowers (N) as a function of N as follows:

$${ROBBED}_{i}=Binomial\left(p_{i}^{\left(R\right)},N_{i}\right),$$

$$logit(p_{i}^{\left(R\right)})=\beta_{i}^{\left(R\right)}+\beta_{2}^{\left(R\right)}\times N_{i}+r_{j}^{\left(R\right)}$$

The expressions above include a random effect for sampling individuals *j*, where *r* ^*(R)*^_*j*_ ~ Normal (0, σ_R r_). The term σ_R r_ represents the standard deviation of *r* ^*(R)*^_*j*_.

Then, we modeled the number of damaged styles (D) in inflorescent *i* as a function of ROBBED as follows:

$$D_{i}=Binomial\left(p_{i}^{\left(D\right)},{ROBBED}_{i}\right),$$

$$logit(p_{i}^{\left(D\right)})=\beta_{1}^{\left(D\right)}+\beta_{2}^{\left(D\right)}\times ROBBED_{i}+r_{j}^{\left(D\right)}$$

The expressions above include a random effect for sampling individuals *j*, where *r* ^*(D)*^_*j*_ ~ Normal (0, σ_D r_). The term σ_D r_ represents the standard deviation of *r* ^*(D)*^_*j*_.

#### Pollinator visitation rates

We modeled the number of pollinator visitations (V) for individual plant *i*, as a function of pollinators (POLL: two levels, *Apis cerana* and *Bombus eximius*), robbing (ROBBED: two levels, robbed and unrobbed), and its interaction, as follows:

$$V_{i}=Poisson\left(\lambda_{i}^{\left(V\right)}\right),$$

$$\log\left(\lambda_{i}^{\left(V\right)}\right)=\beta_{1}^{\left(V\right)}+\beta_{2}^{\left(V\right)}\times POLL_{i}+\beta_{3}^{\left(V\right)}\times ROBBED_{i}+\beta_{4}^{\left(V\right)}\times POLL_{i}\times ROBBED_{i}+r_{j,k}^{\left(V\right)}$$

The expressions above include a random effect for plot *j* and sampling date *k* simultaneously, where *r* ^*(V)*^_*j*,*k*_ ~ Normal(Σ*μ* ^*(V)*^/*n*_*k*_, σ_V r_), *μ* ^*(V)*^ is the expected value for adjacent to day *k* on a plot *j*, and *n*_*j*_ is the number of days adjacent to day *k* (i,e, 1 or 2). The term σ_V r_ represents the standard deviation of *r* ^*(V)*^_*j*,*k*_. We used conditional autoregressive (CAR) models to control temporal autocorrelation of visitation rate.

We used independent and uninformative prior distributions for the parameters. The marginal posterior distributions of all parameters were estimated using the Markov chain Monte Carlo (MCMC) method, whereby the model simulations were ran in WinBUGS 1.4.3 [43]. For each model, a thinning rate of 20 was applied and three independent chains of 10,000 length were ran, discarding the first 2,000 as burn-in. This resulted in a posterior distribution consisting of 1,200 samples for each parameter in each model. We used these samples to calculate the mean, standard deviation, and 95% confidence intervals of parameter estimates from the posterior distribution. We assessed model convergence by applying Gelman-Rubin convergence diagnostic [44] with all diagnostic values set at < 1.1. All statistical analyses were conducted in the programming and statistical language using R Core software version 2.13.2. [45].

## Results

### Effects of nectar-robbing on pollen transfer

**Fig 3:**
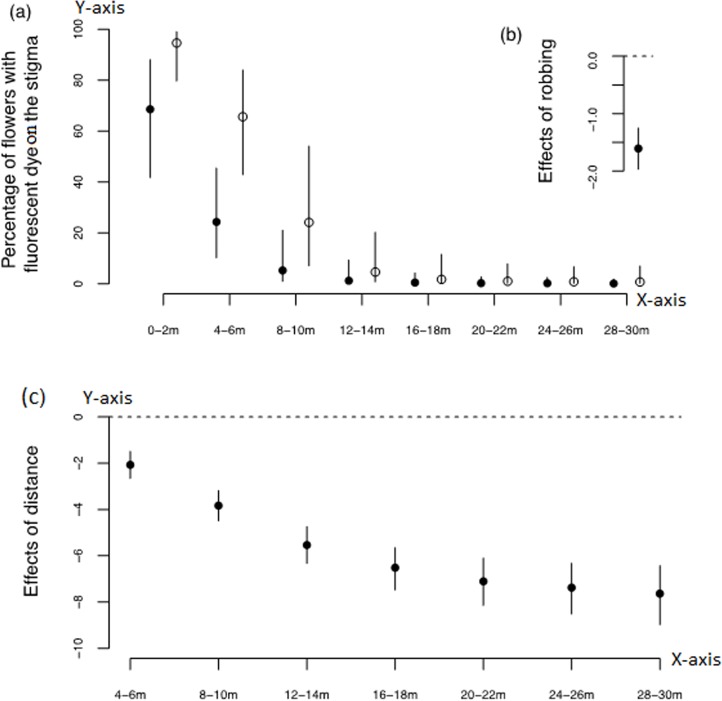
(a) Posterior means of florescent powder dispersal probability (%) among robbed (closed circles) and unrobbed (open circles) plots. (b) Effects of robbing on florescent powder dispersal probability (c) Effects of distances on florescent powder dispersal probability. The effects of distances were calculated with respect to that measured for 0–2 m (i.e., the effect for 0–2 m was set to zero and was used as a benchmark). Bars represent the 95% confidence interval of the posterior distribution in each figure.

Pollen dispersal decreased with increasing distance and was lower in robbed FDP compared to unrobbed FDP (Fig 3A; Table 1). The 95% confidence showed that the effects of distance did not included zero and these effects increased until 12–14 m (Fig 3B), indicating that pollen dispersal significantly decreased with increasing distance among all four directions due to squirrel robbing effects (Table 1; Fig 3C). The mean value of pollen dispersal distance was 0.27 m ± 0.03 (std.error) (8 days, mean ranges 1–30 m) in unrobbed plots and 0.14 m ± 0.05 (std.error) (8 days, mean ranges 1–20 m) in robbed plots (S1 Table). The percentage frequency of pollen transfer distance as affected by squirrel robbing was as follows: W-E direction; 13% of pollen dispersed from robbed plot and 24% from unrobbed plot, N-S direction; 0.8% of pollen dispersed from robbed plot and 31% from unrobbed plot, E-W direction; 18% of pollen dispersed from robbed plot and 26% from unrobbed plot, and S-N direction; 17% and 25% of pollen transferred from robbed and unrobbed plots, respectively. This indicates that squirrel robbing behaviour influenced pollen transfer distance and *A*. *roxburghii* male reproductive fitness.

**Table 1:** Effect of striped-squirrel robbing behaviour on pollen dispersal at various distances. Distances represent 2 m intervals from the focal point.

	Mean	SD	0.025	0.975
Intercept	2.621	0.331	**1.996**	**3.273**
Robbing:Robbed	-1.608	0.185	**-1.975**	**-1.250**
Distance:4–6m	-2.065	0.300	**-2.665**	**-1.481**
Distance:8–10m	-3.831	0.347	**-4.505**	**-3.177**
Distance:12–14m	-5.532	0.402	**-6.335**	**-4.736**
Distance:16–18m	-6.510	0.467	**-7.482**	**-5.643**
Distance:20–22m	-7.106	0.516	**-8.155**	**-6.095**
Distance:24–26m	-7.376	0.573	**-8.523**	**-6.317**
Distance:28–30m	-7.636	0.643	**-8.987**	**-6.420**

Predictor variables, means, standard deviation (SD) and 95% confidence intervals are indicated. Means were calculated from posterior distributions. Bold values are significantly different from zero (based on 95% confidence intervals). The effects of robbing were calculated with respect to unrobbed (i.e, the effect of unrobbed was set to zero). The effects of distances were calculated with respect to that for 0–2m (i.e, the effect for 0–2m was set to zero).

### Effects of nectar-robbing on fruit and seed set

**Fig 4:**
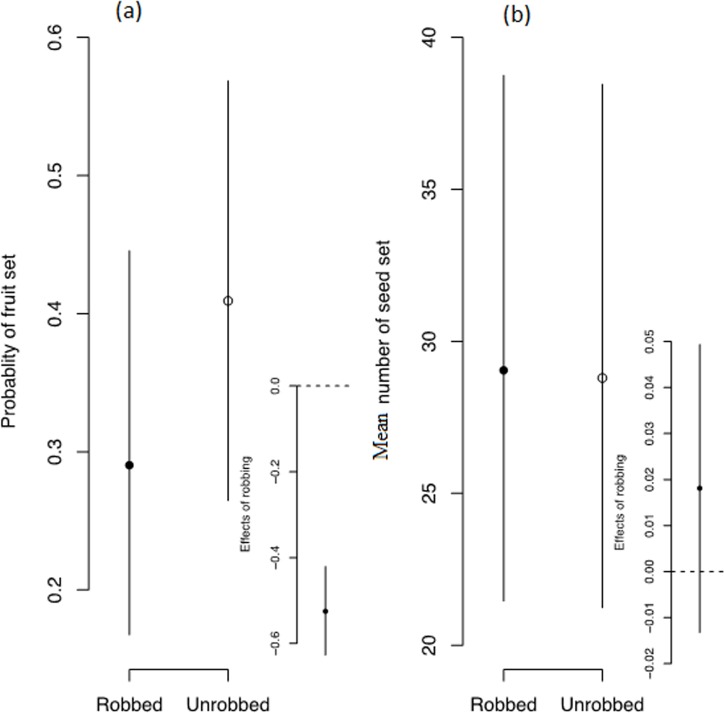
Effect of nectar-robbers on (a) fruit and (b) seed set in *Alpinia roxburghii*. Open and closed circles represent posterior means and bars represent the 95% confidence interval of the posterior distribution.

In robbed FDP, 30.03% of fruits were recorded (5174 fruits/17231 flowers), whereas 41.6% of fruits (7001 fruits/16845 flowers) were recorded in unrobbed FDPs for two years (2005 and 2007) and for a total of 240 inflorescences in each FDP (S2 Table). Fruit set was significantly higher in unrobbed FDP than in robbed FDP (Fig 4A, Table 2). The percentage of seed set was 28.94% (25502 seeds/881 fruits) in robbed FDP, and 29.67% (24159 seeds/814 fruits) in unrobbed FDP, a 0.73% reduction in seed set in robbed FDP as compared to unrobbed FDP (S2 Table). This indicates that the effects of robbing substantially reduces fruit set but does not affect seed set (Fig 4B, Table 2).

**Table 2:** Summary of robbing effects on fruit and seed set. The parameters were calculated from the posterior probabilities of the model parameters.

	Fruit-set				Seed set			
	Mean	SD	0.025	0.975	Mean	SD	0.025	0.975
Intercept	-0.369	0.130	**-0.626**	**-0.124**	3.348	0.038	**3.270**	**3.413**
Robbing: Robbed	-0.525	0.052	**-0.628**	**-0.420**	0.018	0.016	-0.013	0.049

Predictor variables, means, standard deviation (SD) and 95% confidence intervals are indicated. Means were calculated from posterior distributions. Bold values are significantly different from zero (based on 95% confidence intervals). The effects of robbing were calculated with the respect to unrobbed (i.e, the effects of unrobbed was set to zero).

### Floral damage caused by the striped-squirrel

**Fig 5:**
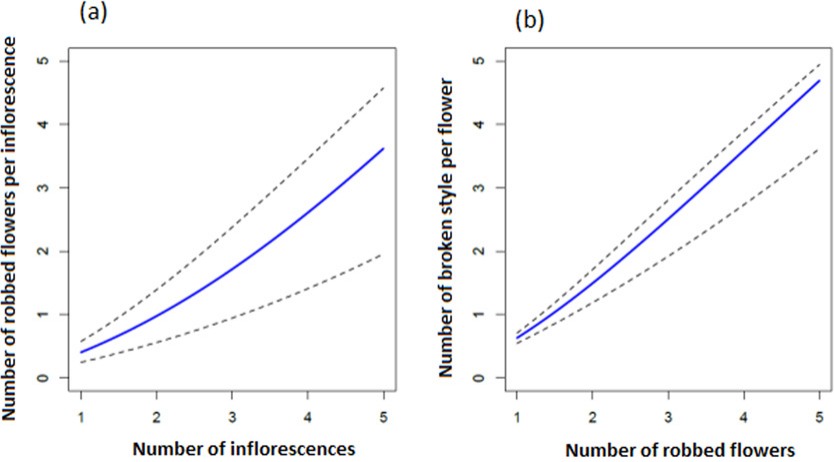
The relationship between (a) number of inflorescences and number of robbed flowers per inflorescence (b) number of robbed flowers and number of broken styles per robbed flowers by striped- squirrels.

Squirrels first make a hole at the base of the corolla, then drink the nectar, damaging the style and stamens but normally leaving the flower relatively intact (recorded in 60% of cases in robbed FDP; Fig 1A). In 40% of cases, the flower was completely removed and discarded after nectar had been consumed (Fig 1B), according to the video recordings and field observations. In each day, 46–118 flowers were robbed and 39–98 flowers had their styles broken by striped-squirrels. The total number of robbed flowers was 458 and the total number of broken styles was 365 over five consecutive days. In addition, mean number of robbed flowers was 91.6 ± 12.81 (mean ± std. error) and mean number of flowers with broken styles was 73 ± 9.88 (mean ± std. error) for four hours visits/day. The frequency of damaged styles increased significantly with increasing number of robbed flowers (Fig 5A and 5B; Table 3). Consequently, striped-squirrel robbing behaviour significantly affects male and female floral parts through direct effects on floral damage.

**Table 3:** Effect of striped-squirrels robbing on broken styles.

	Mean	SD	0.025	0.975
Intercept	-2.487	0.475	**-3.374**	**-1.556**
Robbing: Robbed	1.347	0.158	**1.071**	**1.686**

Predictor variables, means, standard deviation (SD) and 95% confidence intervals are indicated. 95% confidence intervals in bold type indicates significant effects. The effects of robbing was calculated with respect to unrobbed (i.e, the effect of unrobbed was set to zero).

### Flower visitors

**Fig 6:**
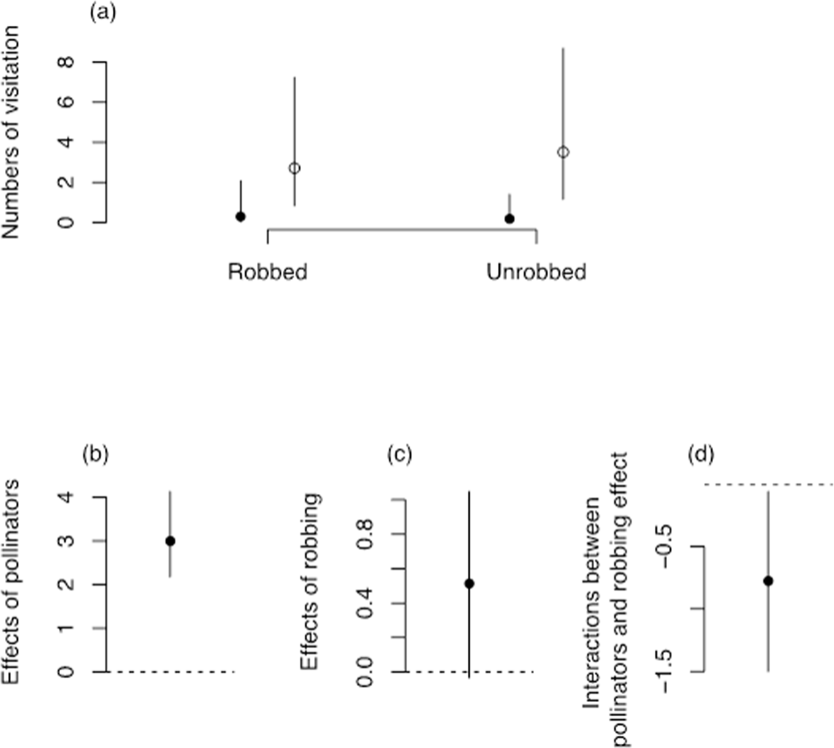
(A) Posterior means of numbers of *Bombus eximius* (open circles) and *Apis cerana* (closed circles) between robbed and unrobbed plots. (B) Effects of pollinators indicates the effect of *Apis cerana* was set to zero. (C) Effects of robbing. (D) Interactions of robbing and pollinators, and their effects. Bars represent the 95% confidence interval of the posterior distribution in each figure.

The visitation rates of *B*. *eximius* and *A*. *cerana* were different (Fig 6A), with *B*. *eximius* visiting more often than *A*. *cerana*. The interaction between *‘B*. *eximius’* and ‘robbing’ was negative (Fig 6C, Table 4), which indicates that the difference in visitation rate by *B*. *eximius* decreased in robbed and increased in unrobbed FDPs (Fig 6A). In contrast, *Apis cerana* visitation rate increased in robbed and decreased in unrobbed FDPs (Fig 6D, Table 4). The pollinator visitation rate of *B*. *eximius* was 6.15 ± 0.82 and 7.86 ± 0.94 (mean ± std. error) visits per hour, whereas *A*. *cerana* was 3.06 ± 0.81 and 1.33 ± 0.34 (mean ± std. error) visits per hour in robbed and unrobbed FDPs respectively.

**Table 4:** Pollinator visitation rates with respect to robbing between robbed and unrobbed FDPs.

	Mean	SD	0.025	0.975
Intercept	-1.742	0.536	**-2.903**	**-0.912**
Pollinator: *B*.*eximius*	2.999	0.516	**2.169**	**4.135**
Robbing: Robbing	0.514	0.286	-0.036	1.049
Pollinator: *B*. *eximius;* Robbing: Robbing	-0.777	0.365	**-1.504**	**-0.059**

Predictor variables, means, standard deviation (SD) and 95% confidence intervals are indicated. Mean were calculated from posterior distributions. Bold values are significantly different from zero (based on 95% confidence intervals). The effects of pollinator visits (robbed plots) were calculated with respect to *A*. *cerana*. (i.e., the effect of *A*. *cerana* was set to zero). The impact of robbing was calculated with respect to unrobbed (i.e, the effect of unrobbed was set to zero).

## Discussion

Our results suggest that pollinator visitation rate differed between robbed and unrobbed FDPs and demonstrated that *B*. *eximius* visitation rate was higher than that of *A*. *cerana* for both robbed and unrobbed FDPs. These constrasting nectar robbing activities may influence pollinator visitation rates and decrease fruit set, but not seed number per mature fruit. We also examined how nectar robbing affected male and female reproductive success. Pollen dispersal distance was lower in robbed than in unrobbed FDP, indicating that nectar theft influences pollen flow activity. Similarly, fruit set significantly decreased in robbed FDP whereas seed set was not significantly affected. This result indicates that a full seed set was achieved in few visits, through the effieciency of *Bombus* as a pollinator. Although squirrel-nectar robbing behaviour directly damages the floral parts of plants, this does not affect seed set and female reproductive fitness of intact flowers.

Previous studies find that *A*. *roxburghii* flowers lasts only one day (12 h), and produce nectar throughout the day [33]. Since nectar availability determines the visitation rate and reproductive success of flowers, nectar theft floral damage limits the availability of nectar to legitimate pollinators [20, 19, 17].

### Squirrel damage to flowers

Our observations revealed substantial damage was caused by squirrels to flowers and other floral parts. Two main types of nectar-robbing behaviours were observed in the study; partial damage to floral structures (thereby potentially decreasing reproductive success), and complete removal of whole flowers (therefore preventing reproduction). In the former behaviour (which occurred on 60% occasions) squirrels made a hole at the base of the corolla, then drank the nectar, sometimes damaging the style and stamens but normally leaving the flower relatively intact. In the latter case (40% of theft events), the flower was completely removed, and discarded after nectar had been extracted [8]. Squirrels did not eat the flowers while robbing nectar according to our observations.

Interestingly, robbing generally occurred between 8–10 AM, and occasionally between 2–4 PM, when the majority of bee visits to flowers occurred. This implies that the damage to flowers occur before any opportunity for successful pollination. In this region of subtropical China, two species of *Alpinia* co-occur, both of which were targeted by squirrels. However, though *A*. *roxburghii* (which covered over 50 ha) was largely targeted from 8–10 AM, all *A*. *kwangsiensis* (covering under 1 ha) were -robbed by squirrels by 7 AM, and therefore either open earlier or are a preferred food source for squirrels.

Given that nectar-robbing occurs before legitimate pollinators are active, the visible damage to flowers may decrease attractiveness and therefore pollination by pollinators. This may lead to discrimination against robbed flowers and the high level of nectar-robbing may affect the abundance of legitimate pollinators in the entire robbed FDP.

The case of the striped-squirrel is resembles that of *Curaeus curaeus* (Austral blackbird) on *Puya coerulea* [20] in which *C*. *curaeus* causes similar damage to robbed flowers, and has similar effects in reducing visitation rates by legitimate pollinators and in successful fruit set and seed production. It is likely that vertebrate nectar-robbers have greater negative effects on plant reproductive success than invertebrate robbers, because they are generally less well suited to legitimately acquire nectar or transfer pollen between plants, and therefore must cause significant damage by robbing behaviour. Plants such as gingers are generally robust enough to provide access to both mammalian and avian potential robbers, and are therefore more likely to be the victims of nectar theft than more fragile flowers.

### Pollinator behaviour

**Fig 7:**
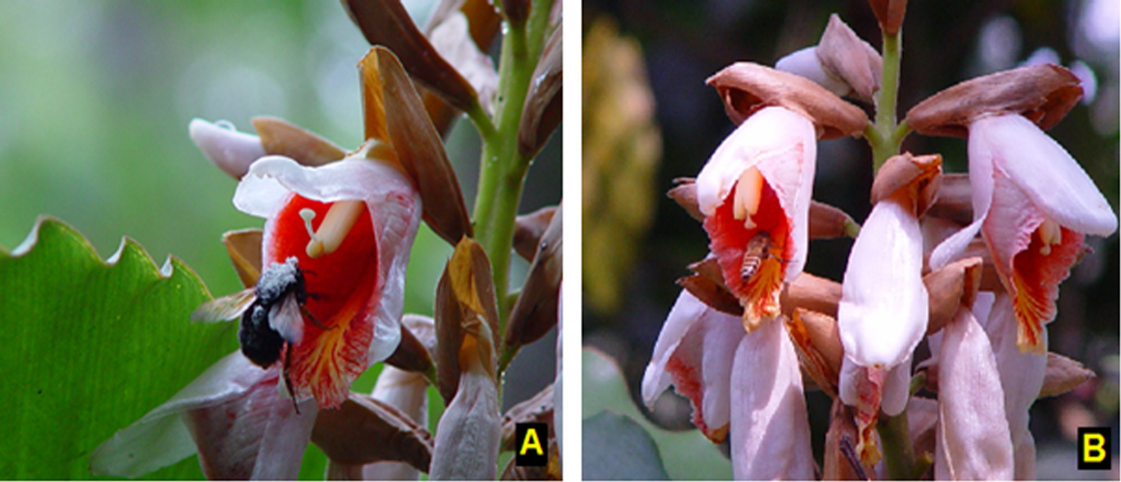
Main pollinators (A) *Bombus eximius and* (B) *Apis cerana* pollinating *A*. *roxburghii* flowers.

The main pollinator within the study was *B*. *eximius* (Fig 7A), with *A*. *cerana* also having a significant but lesser role in successful pollination (Fig 7B). When it encounters unrobbed flowers, *B eximius* travels greater distances within a plot and thereby increase pollen transfer and pollen transfer distance, whereas *B eximius* is more likely to leave after encountering a robbed flowers [7]. Clearly given the level of damage by squirrels on robbed FDP (59–91% of flowers), leaving the plot is likely to decrease the probability that *B*. *eximius* will encounter further damaged flowers because damage is likely to reduce nectar reward and availability. The mean visitation rate of *B*. *eximius* was also lower in robbed FDP that visited an unrobbed FDP in the same area, indicating that *B*. *eximius* visitation rate was influenced by the availability of robbed flowers.

*Apis cerana* seemed less likely than *B*. *eximius* to detect whether a flower had been robbed, and would frequently visit all flowers within an area (Fig 7B). *Apis cerana* visited flower more in robbed FDP, which may increase the seed sets within robbed plots.

### Male reproductive success impact from squirrel robbing

The quantity of pollen dispersed decreased significantly with increasing distance, and was significantly lower overall in robbed FDP than in unrobbed FDP. This was found in all four directions within the plot, with pollen dispersal increasing more in unrobbed FDP than in robbed FDP. A prior study also shows the same pattern of pollen flow distance, reduced among robbed flowers compared to unrobbed flowers [7].

Previous studies on the impacts of insect and avian nectar-robbers on male reproductive fitness, across a number of tropical and temperate plant species, report inconsistent results which include both positive and negative effects [5, 15, 19, 46].]. The method of nectar removal and the impact on plants varies significantly between species [2]. However, in this study, the effect of striped-squirrel robbing behaviour had substantial negative impacts on male reproductive fitness of *A*. *roxburghii*, through flower damage. The damage to *A*. *roxburghii* male reproductive parts and decreased rate of visitation by the most effective pollinators, are the two main causes of decreased plant reproductive fitness. For instance, distance travelled during foraging may increases energy expenditure, and may further reduces fitness through increased foraging time and exposure to predators. Damage to flowers as a result of robbing prevents normal reproductive function, including pollinator attraction, pollen dispersal, pollen transfer and fertilization.

### Effects of nectar-robbing on fruit and seed set

Few studies have examined reproductive success for male and female flowers together [8, 11]. Although fruit-set in this study was reduced in robbed plots (11.57% fruit reduction), seed-set showed only a relatively small reduction (0.73% decrease), resulting in a net loss of 12.22% potential seeds.

The reproductive success of *A*. *roxburghii* is dependent upon successful pollination. Earlier studies also reported that direct negative impacts of nectar-robbing on fruit set of *A*. *kwangsiensis* [11], and direct negative impact on female reproductive success from avian nectar-robbers [8, 20]. Several studies demonstrate that indirect negative effects influence female reproductive success in a number of species including *Ipomopsis aggregata* [24], *Vitex negundo* [47], *Quassia amara* [48], and *Polygala vayredae* [7]. However in some species, no effects are found, (i.e *Vicia faba*) following nectar theft by bumblebees [49]. A number of genera including *Alpinia* and *Amomum* (Zingiberaceae) [50], exhibit interesting flexistyly polymorphisms [51] which enhance fruit production by avoiding geitonogamy and promoting xenogamy [33, 35]. However, damage from striped-squirrels by removing most of the nectar and cutting styles may remove any potential advantages derived from flexistyly.

Studies also show that fruit-set occurrs under self-compatibility, whereas no fruit set occurred in the unpollinated bagged plants, and therefore no autogamy or apomixis resulting no fruit in the studied species [33, 35]. Since the probability of pollination, coupled with the distance and quantity of pollen transferred was significantly lower in robbed plots, fruit-set may also decrease, as we demonstrated in the experimental plots. What remains unexpected is that seed-set was only reduced by a small amount (28.94% in robbed FDP and 29.67% in unrobbed FDP). This nonsignificant reduction in seed set indicates that high prevalence of robbing on a plant did not significantly affect its seed set, and may in our study be linked to the peristent foraging behaviour of *Apis cerana*, despite robber activity. This is consistent with findings that pollinators can still visit robbed flowers within a short distance, even with minimal nectar availability and/or reward [18, 24], and has no mechanism to detect or avoid the consequences of floral damage or reduced floral rewards. We found that pollinator visits did not differ significantly between robbed and unrobbed FDPs and this provides an explanation for the similar number of seed sets in both FDPs.

## Conclusion

Overall, nectar-robbing by squirrels had both direct and indirect effects on male (pollen-flow distance) and female (fruit set) flowers thus influencing plant reproductive success. Although squirrel robbing behaviour directly damaged the floral parts (by breaking styles) of *A*. *roxburghii*, this did not significantly affect seed set, and suggests that squirrel robbing behaviour has no significant effect on female reproductive fitness. This finding is likely due to single pollinator visits occurring in the early morning before pollen is depleted in male flowers and before robbing has cut the floral style (or altered other floral morphology) of the female flowers, which may negatively influence other flower visitors. However, our study revealed that striped squirrel nectar robbing behaviour significantly impacted the male fitness of *A*. *roxburghii* flowers by negatively affecting pollen-flow distance. However, furthur experimental study would allow us to understand the impact of striped-squirrel nectar robbing on less sensitive pollinators and flower visitors, which may nonetheless visit flowers after they have been damaged through robbing.

## Supporting Information

S1 Table Mean flower visits with fluorescent dye in the stigma of each flower for robbed and unrobbed Four Directional Plots (FDP). (DOC)

S2 Table Data shows that impact of nectar robbing on fruit set based on number of flowers and inflorescences, and seed set based on number of fruits in robbed and unrobbed FDP, during 2005 and 2007. (DOC)
