# Supplementary material for: Impact of Striped-Squirrel Nectar-Robbing Behaviour on Gender Fitness in Alpinia roxburghii Sweet (Zingiberaceae)
Source: PLoS One. 2015 Dec 21;10(12):e0144585. doi: 10.1371/journal.pone.0144585 (PMC4687006; doi:10.1371/journal.pone.0144585)
Supplement: S1 Table — (DOC) [file pone.0144585.s001.doc]

**Supporting information**

**S1 Table.** Mean flower visits with fluorescent dye in the stigma of each flower for robbed and unrobbed Four Directional Plots (FDP). (DOCX).

| FDP distance (m) | Robbed FDP | Unrobbed FDP |
| --- | --- | --- |
|  | Mean flowers visits with fluorescent dye in the stigma | Mean flowers visits with fluorescent dye in the stigma |
| **W-E** |  |  |
| 0-2m | 0.71 | 0.89 |
| 4-6 | 0.16 | 0.75 |
| 8-10 | 0.08 | 0.09 |
| 12-14 | 0.05 | 0.10 |
| 16-18 | 0.01 | 0.07 |
| 20-22 | 0.00 | 0.02 |
| 24-26 | 0.00 | 0.00 |
| 28-30m | 0.00 | 0.00 |
| **N-S** |  |  |
| 0-2m | 0.30 | 0.99 |
| 4-6 | 0.21 | 0.74 |
| 8-10 | 0.05 | 0.52 |
| 12-14 | 0.01 | 0.10 |
| 16-18 | 0.03 | 0.03 |
| 20-22 | 0.01 | 0.06 |
| 24-26 | 0.00 | 0.06 |
| 28-30 | 0.00 | 0.01 |
| **E-W** |  |  |
| 0-2m | 0.76 | 0.91 |
| 4-6 | 0.38 | 0.53 |
| 8-10 | 0.17 | 0.39 |
| 12-14 | 0.05 | 0.11 |
| 16-18 | 0.05 | 0.05 |
| 20-22 | 0.01 | 0.03 |
| 24-26 | 0.03 | 0.00 |
| 28-30 | 0.00 | 0.03 |
| **S-N** |  |  |
| 0-2m | 0.78 | 0.93 |
| 4-6 | 0.41 | 0.56 |
| 8-10 | 0.10 | 0.24 |
| 12-14 | 0.04 | 0.09 |
| 16-18 | 0.00 | 0.00 |
| 20-22 | 0.01 | 0.00 |
| 24-26 | 0.00 | 0.01 |
| 28-30 | 0.01 | 0.14 |
