# Supplementary material for: Impact of Striped-Squirrel Nectar-Robbing Behaviour on Gender Fitness in Alpinia roxburghii Sweet (Zingiberaceae)
Source: PLoS One. 2015 Dec 21;10(12):e0144585. doi: 10.1371/journal.pone.0144585 (PMC4687006; doi:10.1371/journal.pone.0144585)
Supplement: S2 Table — (DOC) [file pone.0144585.s002.doc]

**Supporting information**

**S2 Table.** Data shows that impact of nectar robbing on fruit set based on number of flowers and inflorescences, and seed set based on number of fruits in robbed and unrobbed FDP, during 2005 and 2007. (DOCX)

| Year | Treatment | FDP directions | Total no. of inflorescences | Total no. of flowers | Total no. of fruits | Number of seeds/No. of fruits |
| --- | --- | --- | --- | --- | --- | --- |
| 2005 | Robbed | W-E | 60 | 2080 | 695 | 3419/124 |
|  |  | N-S | 60 | 2234 | 612 | 2991/124 |
|  |  | E-W | 60 | 2766 | 510 | 2572/84 |
|  |  | S-N | 60 | 2031 | 409 | 2606/85 |
| 2005 | Unrobbed | W-E | 60 | 2365 | 816 | 1572/54 |
|  |  | N-S | 60 | 2233 | 820 | 1745/61 |
|  |  | E-W | 60 | 2442 | 907 | 1394/48 |
|  |  | S-N | 60 | 2277 | 880 | 2492/75 |
| 2007 | Robbed | W-E | 60 | 1869 | 731 | 3413/121 |
|  |  | N-S | 60 | 2021 | 649 | 4519/143 |
|  |  | E-W | 60 | 2053 | 742 | 2985/100 |
|  |  | S-N | 60 | 2177 | 826 | 2997/100 |
| 2007 | Unrobbed | W-E | 60 | 1703 | 756 | 3413/121 |
|  |  | N-S | 60 | 1758 | 794 | 3587/120 |
|  |  | E-W | 60 | 2043 | 1079 | 4760/165 |
|  |  | S-N | 60 | 2024 | 949 | 5196/170 |
